# Efficient Modeling of Single Event Transient Effect with Limited Peak Current: Implications for Logic Circuits

**DOI:** 10.3390/mi15070885

**Published:** 2024-07-05

**Authors:** Yujian Wang, Hongliang Lu, Caozhen Yang, Yutao Zhang, Ruxue Yao, Rui Dong, Yuming Zhang

**Affiliations:** Key Laboratory for Wide Band Gap Semiconductor Materials and Devices of Education Ministry, School of Microelectronics, Xidian University, Xi’an 710071, China

**Keywords:** transient current model, soft error rate, TCAD simulation, logic cell, cell layout, SEMT, radiation effects

## Abstract

The problem that the conventional double-exponential transient current model (DE model) can overdrive the circuit, which leads to the overestimation of the soft error rate of the logic cell, is solved. Our work uses a new and accurate model for predicting the soft error rate that brings the soft error rate closer to the actual. The piecewise double-exponential transient current model (PDE model) is chosen, and the accuracy of the model is reflected using the Layout Awareness Single Event Multi Transients Soft Error Rate Calculation tool (LA-SEMT-SER tool). The model can characterize transient current pulses piecewise and limit the peak current magnitude to not exceed the conduction current. TCAD models are constructed from 28 nm process library and cell layouts. The transfer characteristic curves of devices are calibrated, and functional timing verification is performed to ensure the accuracy of the TCAD model. The experimental results show that the PDE model is not only more consistent with TCAD simulation than the DE model in modeling the single event transient currents of the device, but also that the SER calculated by the LA-SEMT-SER tool based on the PDE model has a smaller error than the SER calculated by the LA-SEMT-SER tool based on the DE model.

## 1. Introduction

Space radiation is one of the most important factors affecting the reliability of devices, circuits, and even electronic systems, which brings a considerable challenge to the reliability of devices. Traditionally, space radiation has been thought to affect only the use of electronic devices in space. However, it has been found that it also affects the use of electronic devices on the ground [1]. The effect of space radiation on electronic equipment is often caused by the single event effect (SEE). A single ion in space strikes a sensitive part of a microelectronic device, generating electron–hole pairs, which change the logic state of the logic cell, and ultimately leading to functional failure. Therefore, the study of the single event effect is necessary.

Single event transients (SETs) are an effect of SEE. When an ion strikes a semiconductor device, electron–hole pairs are generated around the heavy ion’s trajectory. In the electric field, the charge will be collected in a very short time and injected into the active device in the form of a current pulse. These resulting SETs can manifest as injection errors in both digital and analog ICs [2,3,4,5,6,7,8,9,10]. Accurate representation of transient currents by transient current source models is the key to describe SET effects, simulate SET effects, and even calculate device failure rates. Therefore, the study of transient current source models is necessary.

With feature size scaling down, the effect of single event multiple transients (SEMTs) [11,12] on the reliability of circuits is necessary to be considered. Modeling studies of new devices are of interest to many researchers and scholars. Baojun Liu et al. [13] proposed an accurate resolution method for SET-deposited charges in FinFETs. Calculating the deposited charges is important for modeling SETs. E. Amat et al. [14] implement gain-cell embedded DRAMs (eDRAMs) based on FinFET devices and compare the effect of different configurations of device dimensions on the robustness for SEU. A. Calomarde et al. [15] present a novel design style that reduces the impact of radiation-induced single event transients (SET) on logic circuits and apply the methodology to 7 nm FinFET technology.

Establishing transient current source models with concise expressions, accuracy, and good adaptability has been the focus of domestic and international research [16,17,18,19,20,21]. Black et al. [18] described a method using dual double-exponential current sources connected in parallel to model the current waveform resulting from a charge collection event for SET circuit simulations. However, the method using dual double-exponential current sources in parallel was modeled for the phenomenon of brief high current peaks followed by sustained low currents in current transients, and these current transients of brief high current peaks followed by sustained low currents are not common.

The ability of the PDE model and the DE model in describing single event transients is compared. The PDE model is embedded into the LA-SEMT-SER tool. A comparison of the transient current sources expressed by the PDE model and the transient current sources expressed by the DE model from the output of the cells and the LA-SEMT-SER tool used to calculate the SER values reflects the characteristics of both models. The reason for this on the one hand is that the DE model overdrives the circuitry, which leads to the overestimation of the soft error rate of the logic cell. On the other hand, the PDE model has a finer description of transient current pulses, and the peak current of the PDE model is not going to exceed the conduction current. To verify the reliability of the experiments, the tool is applied to the NAND and NOR logic cells, and the experimental results show that the results of the soft error rate calculations are in good agreement with the TCAD simulation results (Sentaurus TCAD 2018).

## 2. Background

### 2.1. Motivation

**Figure 1:**
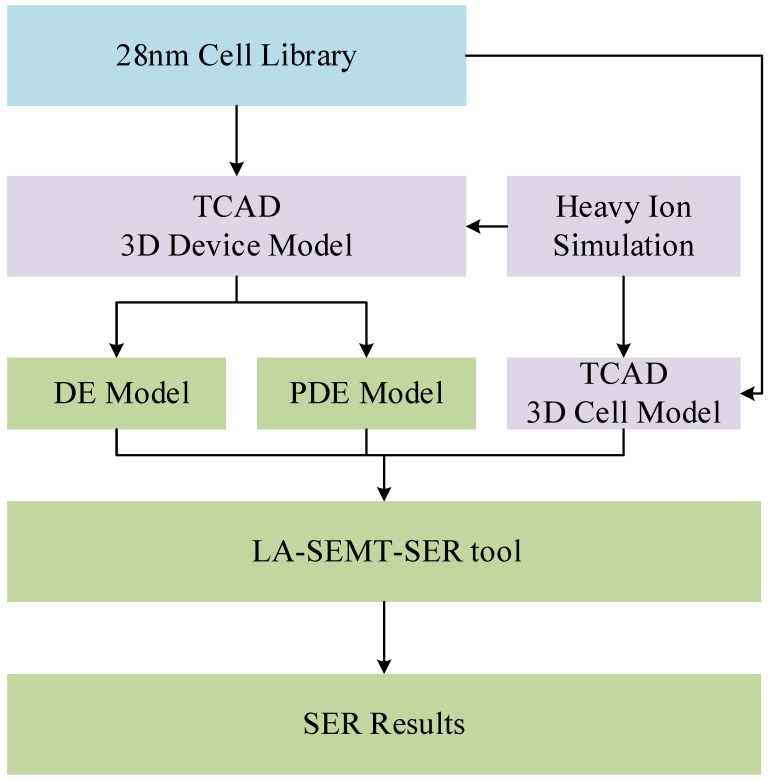
Flowchart of DE model and PDE model using LA-SEMT-SER tool.

Firstly, the PDE model and the DE model have different descriptive capabilities for the transient current generated by the SEE, and the transient current expressions and realizations are different. Secondly, there are some limitations of the multiple transient current pulse models mentioned before. Finally, the transient current pulse models [16,17,18,19] show good properties in different process nodes and different platforms, but the accuracy and generalization of different transient current pulse models in the same process node and in the calculation results using the same tool are yet to be investigated. For the DE model and the PDE model, the results of running the LA-SEMT-SER tool illustrate the accuracy and generalization of the models by comparing them with the TCAD simulation results. The significance of our work is to embed the PDE model into the LA-SEMT-SER tool to predict soft error rates and to compare it with the DE model to reflect the accuracy of the PDE model. The flow methodology is shown in Figure 1.

### 2.2. Limitation of the Models

Messenger et al. [17] proposed an approximate analytical solution in double-exponential form for funneling phenomena to provide a modeling basis for circuits and systems to analyze the effects in computer codes. However, the DE model may not be able to accurately represent SETs that can be captured by time-sequential circuits [22,23] and certain durations that are sufficiently long. In addition, the DE model may overdrive the circuit, resulting in an independent current source that will force the NMOS drain below the ground or VSS voltage rails, thereby overestimating the robustness of the circuit.

Kauppila et al. [16] proposed a bias-based single event model consisting of four equations for calculating bias-dependent SET currents. However, the bias state-based SET current model integrates it in the BSIM4 model, and the Cadence 90 nm Bulk CMOS PDK is difficult to inject into the sensitive region of the device with a separate current source, and its expression is too complex; finally, the model is poorly generalized and requires re-extraction of CS, GREC, and GSEE for different processes.

Black et al. [18], in 2015, described a method using dual double-exponential current sources connected in parallel to model the current waveform resulting from a charge collection event for SET circuit simulations. While the dual double-exponential current sources method is more accurate than double-exponential current sources, the method still suffers from overdriving the output, resulting in a slight overestimation of the amount of charge generated. In addition, the dual double-exponential current sources method works well for longer SET current transients, but it works poorly for shorter SET current transients, which are more relevant for short current pulses that are more likely to occur in a space environment.

Warren et al. [19] used a piecewise exponential current source to identify SEU regions and predict single-event cross sections and soft error rates. The PDE model sources address the situation where previous transient current source methods can cause the circuit overdrive output to drop below VSS. However, the PDE model sources require judgment of the magnitude of the peak value.

PWL models, lookup table (LUT) models, and other current source models all suffer from limitations such as the need for a large amount of simulation or experimental data to support them and their inability to be used for circuit level fault injection.

### 2.3. Transient Model

The DE model and the PDE model used in this experiment and the two transient current models are described in detail below. The transient current pulse is generated according to the drift and diffusion mechanism, and the resulting current transient pulse is usually modeled by the following double-exponential transient current model [24], where the two exponential functions in the expression (1) represent the upward and downward trends of the transient current pulse, respectively:

(1) $$I(t)=\frac{Q}{\tau_{\alpha }-\tau_{\beta }}\left(e^{-t/\tau_{\alpha }}-e^{-t/\tau_{\beta }}\right)$$

where *Q* is the ion impact that produces the charge being collected, *τ_α_* is the collection time constant of the p–n junction, and *τ_β_* is the ion orbital establishment time constant. The time constants *τ_α_* and *τ_β_* depend on the process node.

**Figure 2:**
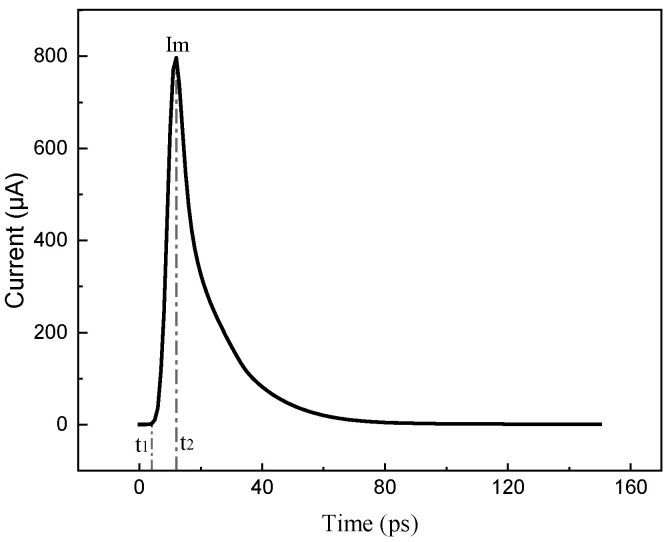
Three parts of the PDE transient current model.

The PDE model differs from the double-exponential model in that it specifies that the maximum value of the current will not exceed the conduction current of the device; thus, the PDE model does not suffer from overdrive output. The PDE model divides the transient current waveform over time into three regions, each of which is shown in Figure 2.

The three curves can be expressed by the following equation [14]:

(2) $$I\left(t\right)=\left\{\begin{array}{ll} 0 & ,\;t\leq t_{1}\\ I_{m}\left(1-e^{-\left(t-t_{1}\right)/\tau_{1}}\right) & ,\;t_{1}<t\leq t_{2}\\ I_{m}e^{-\left(t-t_{2}\right)/\tau_{2}} & ,\;t>t_{2} \end{array}\right.$$

(3) $$Q_{coll}=\int_{0}^{\infty }I\left(t\right)dt=\int_{t_{1}}^{t_{2}}I_{m}\left(1-e^{-\left(t-t_{1}\right)/\tau_{1}}\right)dt+\int_{t_{2}}^{\infty }I_{m}e^{-\left(t-t_{2}\right)/\tau_{2}}dt=Q_{1}+Q_{2}$$

where *t* is the time, *t*_1_ is the pulse delay time, *t*_2_ is the onset of pulse decay, *τ*_1_ is the rise time, *τ*_2_ is the decay time, *I*_m_ is the peak current, and *Q_coll_* is the collected charge from the SEE.

The calculation of the peak current *I*_m_ will be divided into two cases:

(4) $$Q_{coll}<\frac{VDD}{R_{on}}\left(\left(\tau_{1}+\tau_{2}\right)+\left(t_{2}-t_{1}\right)-\tau_{1}e^{-\left(t_{2}-t_{1}\right)/\tau_{1}}\right)$$

If the collected charge *Q_coll_* satisfies Equation (4), then there are:

(5) $$I_{m}=\frac{Q_{coll}}{\left(\tau_{1}+\tau_{2}\right)+\left(t_{2}-t_{1}\right)-\tau_{1}e^{-\left(t_{2}-t_{1}\right)/\tau_{1}}}$$

If the collected charge *Q_coll_* does not satisfy Equation (4), then there are

(6) $$\begin{array}{l} I_{m}=\frac{VDD}{R_{on}}\\ f=\frac{Q_{1}}{Q_{coll}}\\ \tau_{2}=\left(1-f\right)\frac{Q_{coll}}{I_{m}}\\ \left(t_{2}-t_{1}\right)-\frac{Q_{coll}}{I_{m}}+\tau_{1}\left(e^{-\left(t_{2}-t_{1}\right)/\tau_{1}}-1\right)=0 \end{array}$$

*t*_1_ is the pulse delay time, *t*_2_ is the onset of pulse decay, *R*_on_ is the conduction resistancen and *VDD* is the supply voltage. *t*_1_, *t*_2_, *R*_on_, and *VDD* are derived from TCAD device-level simulations of a single NMOS; *τ*_1_ and *τ*_2_ are derived from device-level simulations of the heavy ion model by a Matlab fitting (Matlab 2019b).

## 3. Injection of Transient Model Source

An accurate transient current source is the basis of the circuit-level SEE study; the transistor subjected to ion impact is called the sensitive transistor in the circuit, and a transient current source is added between the drain of the device and the *GND*, so as to realize the simulation of the SET effect; the other transistors in the circuit are modeled by the conventional SPICE transistors, and a joint simulation platform is constructed by combining with the SPICE software (SPICE 2016).

In our work, a 28-nm MOSFET device is used for device-level TCAD heavy ion simulations, with the distance between the primary and secondary devices ranging from 10 μm to 70 μm, the ion incidence angle ranging from 0° to 60°, and the LET of the ions ranging from 0.5 MeV·cm^2^/mg to 100 MeV·cm^2^/mg [25]; and the collected charge *Q_coll_* is calculated by Equation (7).

(7) $$Q_{coll}=F(D,Angle,LET)$$

**Figure 3:**
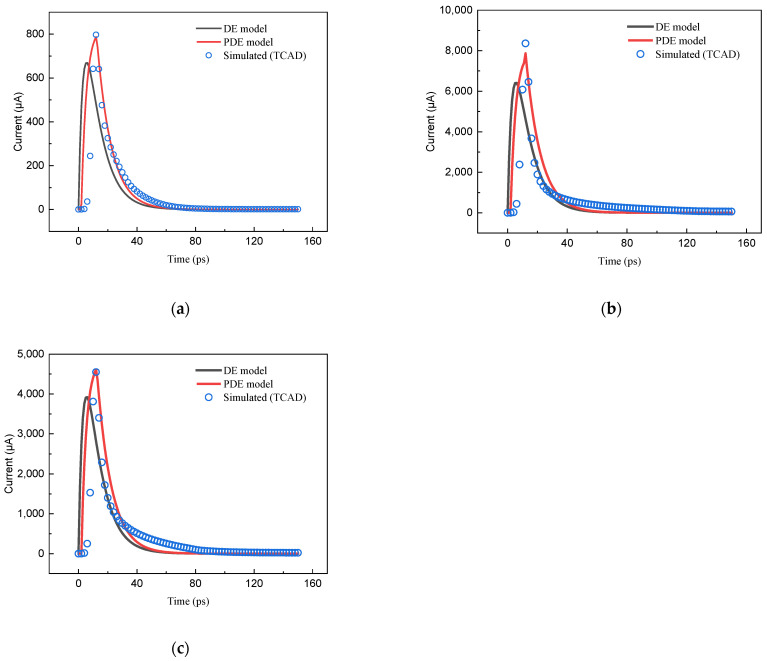
PDE model and DE model describing the TCAD simulation waveforms at *t*_1_ of 2 ps, *t*_2_ of 11.9 ps, *τ*_1_ of 0.4 ps, *τ*_2_ of 1 ps, *τ*_α_ of 3.4 ps, and *τ*_β_ of 10 ps. (**a**) Ion LET is 5 MeV·cm^2^/mg, angle of incidence is 0°, collected charge *Q_coll_* of 11.69 fF; (**b**) Ion LET is 100 MeV·cm^2^/mg, angle of incidence is 30°, collected charge *Q_coll_* is 112.12 fF; (**c**) Ion LET is 40 MeV·cm^2^/mg, angle of incidence is 15°, collected charge *Q_coll_* is 68.58 fF.

From the TCAD simulation, we receive *t*_1_ = 2 pS, *t*_2_ = 11.9 pS, *R*_on_ = 58.935 Ω, and *VDD* = 0.9 V. After the Matlab fitting, then we have *τ*_α_ = 3.4 pS and *τ*_β_ = 10 pS. The waveforms of the transient current from the TCAD simulation, the DE model, and the PDE model are shown in Figure 3. From Figure 3a–c, it can be seen that the PDE model has a more accurate shape in the description of transient pulses than the DE model.

The circuit-level fault injection model is constructed based on Verilog-A, which is a high-level hardware description language for analog circuits. The circuit-level fault injection model based on Verilog-A is continuously varying in current, which is more capable of describing the SET behavior of the ions hitting the sensitive region of the device. In addition, the Verilog-A based circuit-level fault injection model can be mapped to a netlist, so it is compatible with SPICE simulators.

**Figure 4:**
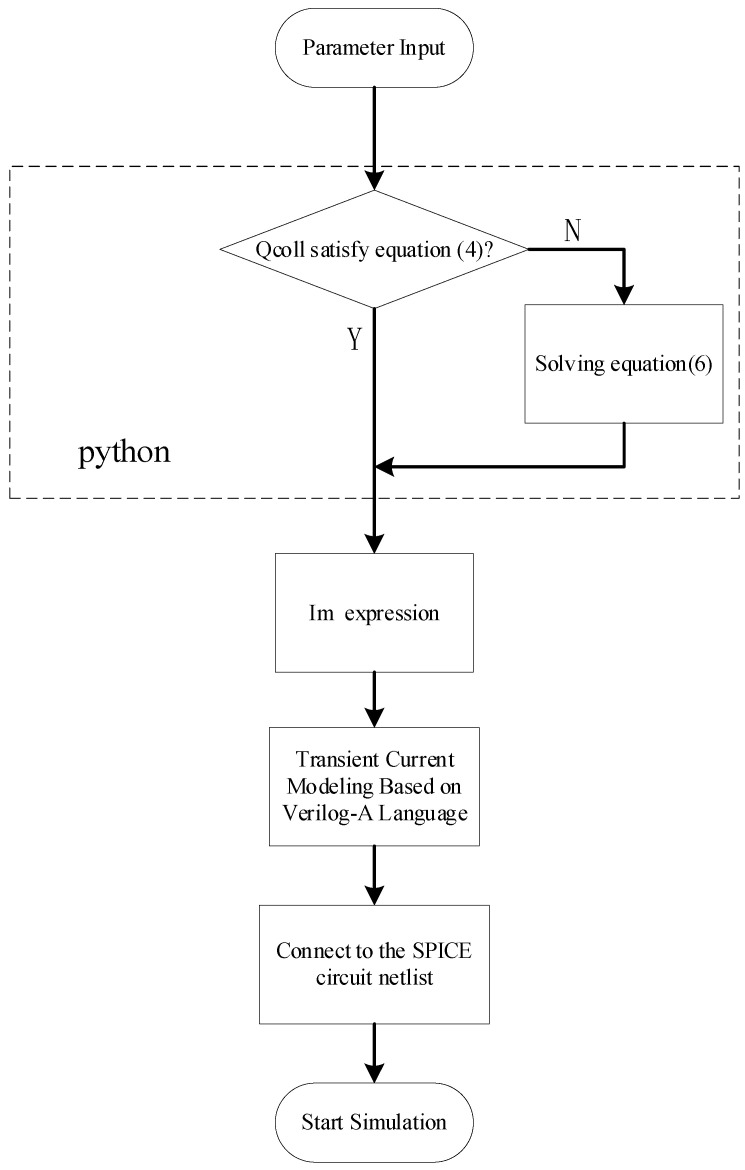
Transient current model embedding based on LA-SEMT-SER tool flowchart.

The *t*_1_, *t*_2_, *τ*_1_, *τ*_2_, *τ*_α_, *τ*_β_, *R*_on_, *VDD*, and *Q_coll_* parameters are entered into the program. The results of *Q_coll_* and Equation (4) are first compared and then the expression for the peak current *I*_m_ is determined. The PDE model is described using the Verilog-A language, and finally the model described by the Verilog-A language is written to the SPICE netlist. The flow of the methodology for embedding the PDE model into the LA-SEMT-SER tool is shown in Figure 4.

## 4. Validation to Experimental Data for CMOS Combinational Cells

**Figure 5:**
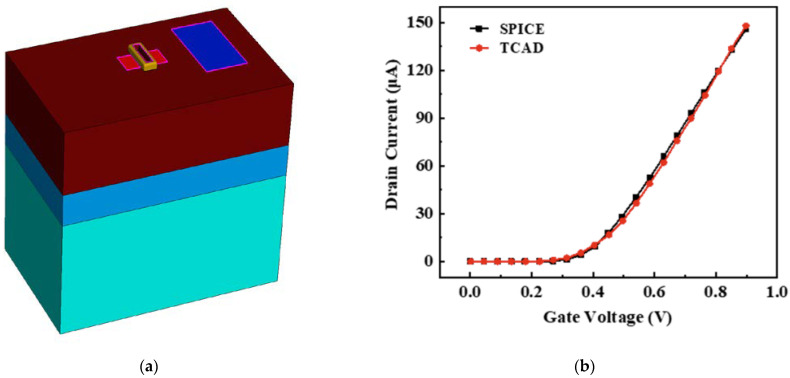
Device level transistor model of 28 nm MOSFET (**a**) 3D NMOS model; (**b**) the calibration of the transfer characteristic curve.

The key to verifying the accuracy of the CMOS combinational cells lies in the establishment of the device-level transistors and the calibration of the transfer characteristic curves. In Figure 5a, the NMOS transistor is simulated in a TCAD based on the 28 nm process library information. It has a length of 0.03 µm and a width of 0.249 µm. In Figure 5b, the transfer characteristic curve of the NMOS transistor is simulated according to SPICE, which makes the TCAD simulated NMOS transistor conform to the 28 nm process library.

**Figure 6:**
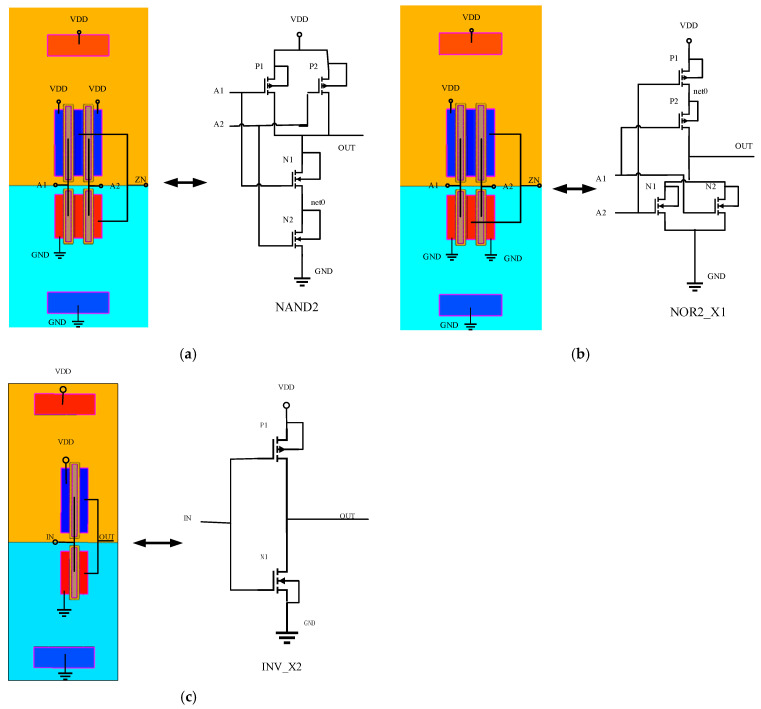
Device model and contact connection of layout (**a**) NAND logic cell; (**b**) NOR logic cell; (**c**) INV logic cell.

The devices can be determined based on the input–output relationship of the cells and the driving strength, while the layout design rules ensure that the constructed TCAD 3D model is realistic. The TCAD 3D model is shown in Figure 6. The 3D TCAD model simulation consumes huge computational efficiency and computational time, and only the cells with a lesser number of transistors and a simple structure are selected for simulation. The NAND and NOR cells in states 00, 01, 10, and 11 are simulated based on the GDS file and PDK library for the 28 nm node. Simulate an INV cell with input states of 0 and 1, and compare the output of an INV cell from the DE model, the PDE model, and the TCAD simulation.

**Figure 7:**
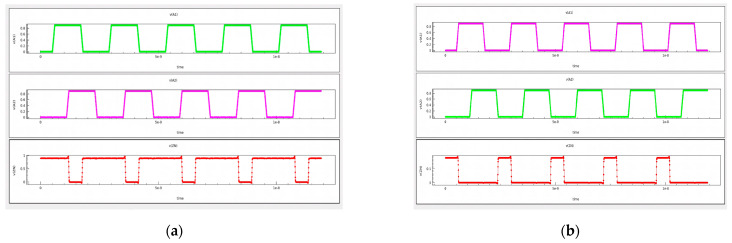
Device model and contact connection of layout (**a**) timing signal analysis of NAND logic cell; (**b**) timing signal analysis of NOR logic cell.

Verification of the layout’s device model and contact connection is subject to timing signal analysis. Functional verification of the cells is necessary to ensure the accuracy of the 3D model being simulated. As shown in Figure 7, the simulated TCAD model satisfies the functions of both NAND and NOR logic cells.

The *LET*_th_ of the logic cells under all inputs are obtained based on a TCAD simulation and a SPICE simulation as shown in Table 1. As can be seen from Table 1, the difference between the *LET*_th_ obtained from the TCAD simulation and the SPICE simulation is small. On the one hand, the TCAD cell-level model can verify the SPICE simulation. On the other hand, the PDE model is able to simulate the single event transient current behavior, which in turn injects the faults into the cells.

**Figure 8:**
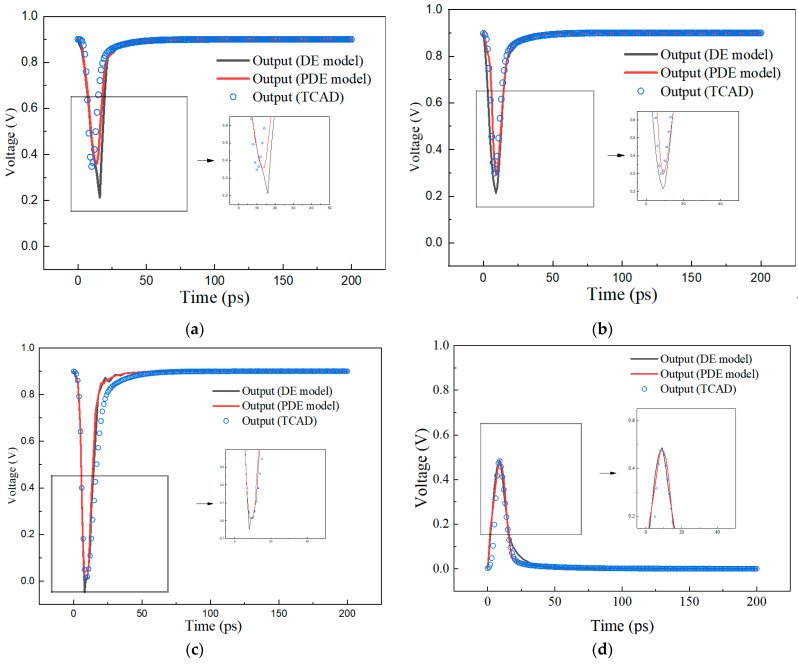

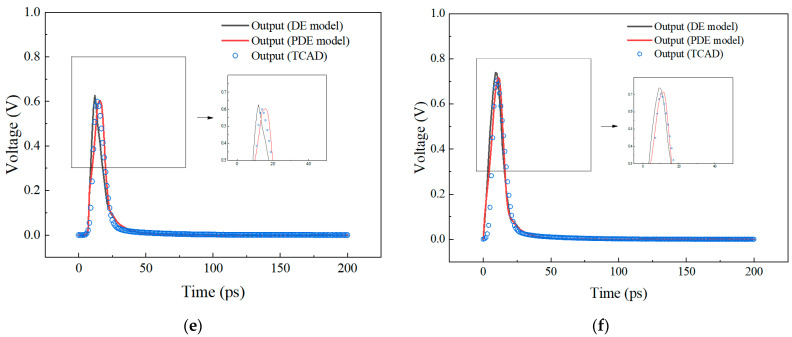
Output waveform of the inverter. (**a**) output = 1, *LET* = 2; (**b**) output = 1, *LET* = 2.12; (**c**) output = 1, *LET* = 3.105; (**d**) output = 0, *LET* = 1.63; (**e**) output = 1, *LET* = 1.85; (**f**) output = 1, *LET* = 2.05.

Injecting a source of current pulses into the sensitive regions of the cell can simulate the behavior of single event transient currents. In the case of an inverter, for example, when the input is low, the NMOS transistor is off, the PMOS transistor is on, and the output is high. At this time, the sensitive region is the drain of the NMOS transistor, and a current pulse source can be connected between the drain of the NMOS transistor and the GND to simulate the particle incidence unit, resulting in output changes. Similarly, when the input is high, the NMOS transistor turns on, the PMOS transistor turns off, and the output is low. At this time the sensitive region is the drain of the PMOS transistor, and a current pulse source can be connected between the PMOS transistor drain and the GND. The inverter output is shown in Figure 8.

The output of the injected PDE model and DE model current sources as well as the inverter output of the TCAD heavy ion simulation are simulated according to the *LET*_th_ of the TCAD and the SPICE. From Figure 8, it can be seen that different LETs produce different errors in the output. The output results of the sensitive node injected with the DE model are higher than the TCAD simulation results. The output results of the sensitive node injected with the PDE model are closer to the TCAD simulation results. Using the DE model injection sensitive node leads to sharp peaks in the output results, which is the DE model overdrive problem mentioned before. This is the reason that using the DE model overestimates the collected charge, which leads to overestimation of the SER of the cell. Using the PDE model does not lead to sharp peaks in the output results, and, therefore, it is more advantageous in estimating the SER of the cell.

After deriving the *LET*_th_ of the cells for all input states, the value of the SER can be determined by calculating the cross section. The SER is calculated by the cross section method. The formula is as follows [26,27]:

(8) $$\begin{array}{l} \sigma =\frac{S}{S_{cell}}\\ \sigma \left(LET\right)=\sigma_{sat}\left\{1-\exp\left[-{\left(\frac{LET-LET_{th}}{w}\right)}^{s}\right]\right\}\\ SER=\int \frac{d\phi \left(LET\right)}{dLET}\sigma \left(LET\right)dLET \end{array}$$

where *S* is the area of the sensitive region, *S_cell_* is the area of the cell, *σ*(*LET*) is the cross section, *σ_sat_* is the saturation cross section area, and *Φ*(*LET*) is the differential flux of the neutron.

The LA-SEMT-SER tool is used to calculate the SER of the cell in the FIT. The tool automatically reads the layout information of the cells, injects the current fault source of the PDE model into the sensitive region, calculates the SER for all the input states, and outputs the final SER. The simulation results are shown in the following table.

In Table 2, the results of the runs using the DE model have an average error of 5.75% when compared to the TCAD simulated NAND2_X1 and NOR2_X1 cell Golden values. The results of the runs using the PDE model have an average error of 2.7% when compared to the NAND2_X1 and NOR2_X1 cell Golden values from the TCAD simulation. The gap between the SER based on the PDE model and the TCAD simulation is smaller, and this gap is 0.28 times of the DE model, which indicates that the PDE model is a more accurate description than the DE model for the transient currents, due to heavy ion incidence on sensitive nodes. The average error of the run results using the PDE model compared to the Golden values of the INV_X2, INV_X4, and INV_X16 cells of the TCAD simulation is 4.7% on the cells with different drive strengths, while the run results using the DE model have an error of 18.9%.

## 5. Discussion

The PDE model has a better ability to express single event transient currents than the DE model. Firstly, in terms of single event transient behavior for a single device, the PDE model is more in line with the TCAD simulation results, and its error is about 15% lower than the DE model. Secondly, the SPICE output of the PDE model as a transient current source indicates that the PDE model can avoid the problem of overdriving the cell output of the DE model, which effectively mitigates the overestimation of the cell’s SER value. Finally, by embedding the PDE model into the LA-SEMT-SER tool, the calculated SER value is within 10% error compared to the cell SER value from the TCAD simulation.

## 6. Conclusions

The LA-SEMT-SER tool based on the PDE model embedded is proposed. The transient current pulse is accurately characterized, and the peak current magnitude is limited to not exceed the conduction current. Single event incidence simulations were performed with a cell model constructed with a TCAD. The LA-SEMT-SER tool was also used to calculate the SER value. The experimental results show that the simulation effect of the PDE model has better superiority compared with the DE model. The PDE model simulates the single event transient current of a single device accurately, and its error with TCAD simulation results is about 15% smaller than that of the DE model. The output curve is smoother when the PDE model is used as a transient current source. The SER value error is smaller when using the PDE model as the LA-SEMT-SER tool.

## Figures and Tables

**Table 1:** The *LET*_th_ (MeV·cm^2^/mg) of the logic cell at all inputs based on TCAD simulation and SPICE simulation.

Cell	Input	TCAD	SPICE
INV	0	2.08	2.12
	1	1.71	1.77

**Table 2:** SER values of cells based on LA-SEMT-SER tool.

Cells	TCAD (Golden)	DE Model (FIT)	Error	PDE Model (FIT)	Error
NAND2_X1	9.9628	10.1722	2.1%	10.1646	2%
NOR2_X1	12.1850	13.3332	9.4%	11.7606	3.4%
INV_X2	1.0451	1.1720	12.1%	1.0142	2.9%
INV_X4	0.2641	0.3039	15.1%	0.2595	1.7%
INV_X16	0.0128	0.0166	29.7%	0.0116	9.4%

## Data Availability

The data presented in this study are available on request from the corresponding author. The data are not publicly available due to the confidentiality of the project.
